# Growth and rupture of pancreatic mucinous cystic neoplasm following hormonal stimulation for *in vitro* fertilization: a case report

**DOI:** 10.1093/jscr/rjaf923

**Published:** 2025-11-24

**Authors:** Italo Simões, Estela Regina Ramos Figueira, Ralph Rodrigo Francisco Martins Tavares, Camila Garcia Marchiolli, José Jukemura

**Affiliations:** Departamento de Gastroenterologia, Faculdade de Medicina da Universidade de São Paulo - São Paulo, Hospital das Clínicas HCFMUSP, Universidade de São Paulo, Av. Dr. Enéas de Carvalho Aguiar, 255, Cerqueira Cesar, CEP 05403-900, Sao Paulo-SP, Brazil; Departamento de Gastroenterologia, Faculdade de Medicina da Universidade de São Paulo - São Paulo, Hospital das Clínicas HCFMUSP, Universidade de São Paulo, Av. Dr. Enéas de Carvalho Aguiar, 255, Cerqueira Cesar, CEP 05403-900, Sao Paulo-SP, Brazil; Departamento de Gastroenterologia, Faculdade de Medicina da Universidade de São Paulo - São Paulo, Hospital das Clínicas HCFMUSP, Universidade de São Paulo, Av. Dr. Enéas de Carvalho Aguiar, 255, Cerqueira Cesar, CEP 05403-900, Sao Paulo-SP, Brazil; Departamento de Gastroenterologia, Faculdade de Medicina da Universidade de São Paulo - São Paulo, Hospital das Clínicas HCFMUSP, Universidade de São Paulo, Av. Dr. Enéas de Carvalho Aguiar, 255, Cerqueira Cesar, CEP 05403-900, Sao Paulo-SP, Brazil; Departamento de Gastroenterologia, Faculdade de Medicina da Universidade de São Paulo - São Paulo, Hospital das Clínicas HCFMUSP, Universidade de São Paulo, Av. Dr. Enéas de Carvalho Aguiar, 255, Cerqueira Cesar, CEP 05403-900, Sao Paulo-SP, Brazil

**Keywords:** mucinous cystic neoplasm, fertilization *in vitro*, pancreas, stromal cells, case report

## Abstract

Mucinous cystic neoplasms (MCNs) of the pancreas are rare mucin-producing epithelial tumors that predominantly affect middle-aged women and typically contain ovarian-type stroma responsive to hormonal stimulation. While progression and rupture of MCNs during pregnancy have been documented, the effects of exogenous hormonal stimulation remain uncertain. We present a unique case of a 39-year-old woman who experienced rapid cystic enlargement and rupture of a pancreatic MCN following hormonal therapy for *in vitro* fertilization (IVF). Imaging demonstrated progressive cyst growth with radiologic evidence of rupture, which was confirmed intraoperatively. Histopathological analysis revealed an MCN with low-grade dysplasia and immunohistochemical positivity for estrogen receptors. This case represents the first description of a potentially severe complication of gonadotropin stimulation in pancreatic MCNs and underscores the importance of screening for cystic pancreatic lesions in patients undergoing assisted reproductive therapies.

## Introduction

In recent decades, reproductive endocrinology and assisted reproductive technologies have advanced substantially. *In vitro* fertilization (IVF) now accounts for 1.6% of all live births in the United States and 4.5% in Europe [1, 2]. IVF protocols involve ovarian stimulation with daily gonadotropin injections, oocyte retrieval, fertilization, embryo culture, and uterine transfer.

Pancreatic mucinous cystic neoplasms (MCNs) are rare epithelial tumors that occur almost exclusively in women, typically between the fourth and sixth decades of life, with an estimated incidence of 1 per 100 000 women annually. These lesions are most often located in the pancreatic body and tail. The epithelial lining may demonstrate low- to high-grade dysplasia or invasive carcinoma, reported in up to 34% of cases [3–5]. Histologically, MCNs are defined by mucin-producing epithelium and ovarian-type stroma, which is usually positive for estrogen and progesterone receptors. This supports a potential hormonal influence on tumor development and helps explain their higher prevalence among middle-aged women [5–7].

Several case reports describe MCN growth, rupture, and hemorrhage during pregnancy—events attributed to pregnancy-associated hormonal fluctuations [8, 9]. However, the effect of exogenous hormonal stimulation, such as that induced by IVF, remains poorly understood, and to date, no cases of MCN rupture following IVF have been documented.

We report a case of a 39-year-old woman who developed progressive enlargement and subsequent rupture of an MCN after multiple cycles of gonadotropin stimulation for oocyte retrieval and cryopreservation. Given the pathophysiological parallels with pregnancy-associated cases, it is plausible that the supraphysiologic hormonal environment induced by IVF played a key role in this presentation.

## Case report

A 39-year-old woman with no comorbidities and no personal or family history of pancreatic neoplasms underwent ovarian stimulation for elective oocyte retrieval and cryopreservation in December 2023. The stimulation protocol included recombinant human follicle-stimulating hormone (FSH) and luteinizing hormone (LH), a gonadotropin-releasing hormone (GnRH) antagonist, and human chorionic gonadotropin (hCG). The procedure was uneventful.

In February 2024, she developed nonspecific epigastric pain, prompting further evaluation. Physical examination was unremarkable. Cardiovascular causes were excluded through exercise stress testing, transthoracic echocardiography, and computed tomography (CT) angiography. Gastroesophageal conditions were ruled out with upper endoscopy and barium esophagography.

Abdominal ultrasonography revealed a well-circumscribed cystic lesion in the pancreatic tail measuring 2.4 × 2.0 × 2.2 cm. The main pancreatic duct was normal, and no gallstones were identified. Contrast-enhanced CT confirmed a unilocular cyst at the body–tail junction of the pancreas (2.1 × 1.8 cm) with an adjacent lobulation (1.2 × 0.7 cm). The posterior wall appeared indistinct, raising suspicion of partial cyst rupture.

**Figure 1:**
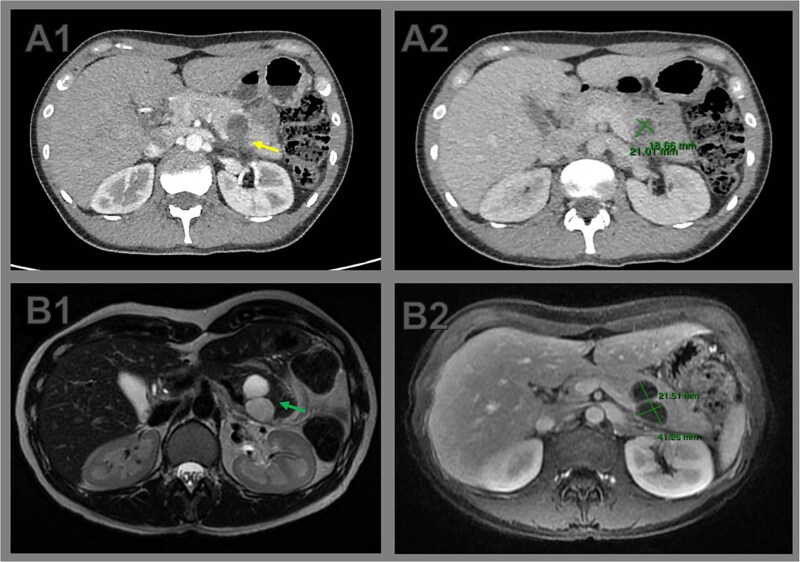
Comparative cross-sectional imaging illustrating progressive growth and signs of rupture in a pancreatic cystic neoplasm. (A1) Axial contrast-enhanced CT image at initial evaluation showing a unilocular cystic lesion at the body–tail junction of the pancreas. The posterior wall appears indistinct (arrow), raising suspicion of partial cyst rupture. (A2) CT measurement of the cystic lesion, with dimensions of 2.1 × 1.8 cm. (B1) Axial T2-weighted MRI performed 2 weeks later reveals a bilobulated cystic lesion in the pancreatic tail, with internal septations (arrow). (B2) Axial T1-weighted MRI shows interval growth of the lesion to 4.0 × 2.1 cm, confirming enlargement and supporting the diagnosis of cyst rupture with associated inflammatory changes.

Two weeks later, the patient developed severe abdominal pain requiring hospitalization. Magnetic resonance imaging (MRI) demonstrated enlargement of the bilobulated cystic lesion to 4.0 × 2.0 cm, with internal septations and thickened contents, consistent with rupture. Associated findings included peripancreatic fat stranding and posterior pararenal fascial thickening. The leading diagnosis was an MCN with partial rupture and secondary pancreatitis; an inflammatory pseudocyst was considered less likely (Fig. 1).

A laparoscopic distal pancreatectomy with splenectomy was indicated, but converted to open surgery due to difficult dissection of the celiac trunk and splenic artery. Postoperative recovery was uneventful. The patient was discharged on postoperative day 5, and drains were removed on day 14 without evidence of pancreatic fistula.

**Figure 2:**
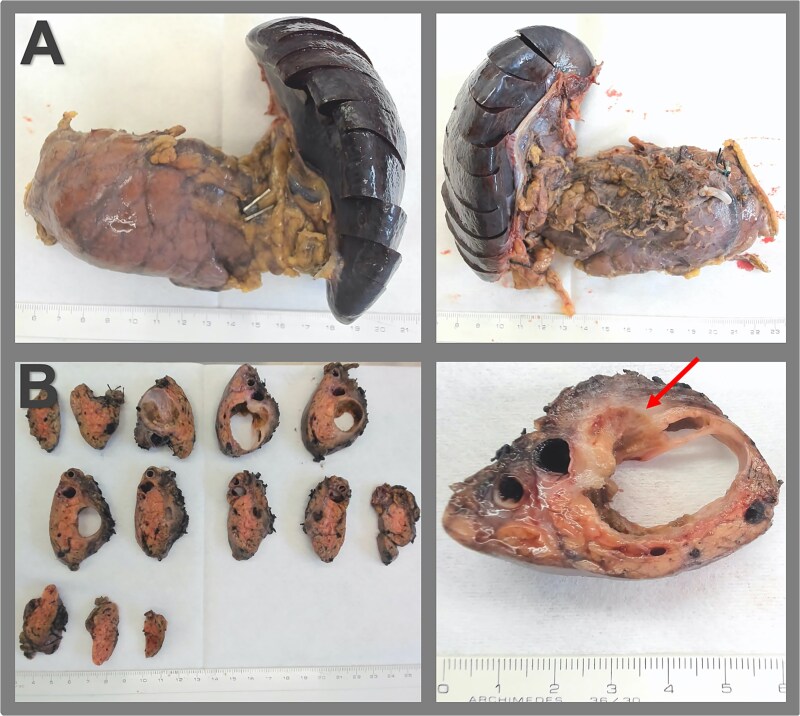
Surgical specimen and macroscopic pathology examination. (A) Specimen of distal pancreatectomy with splenectomy, presented in the anterior and the dorsal view. (B) Macroscopic pathological examination reveals a multiloculated cystic lesion within the pancreas, measuring 3.7 × 2.8 × 2.7 cm, fibrous cyst wall up to 0.2 cm, an adjacent firmer region measuring 3.0 cm extending to the posterior surface of the pancreas, and signs of capsular rupture (arrow).

**Figure 3:**
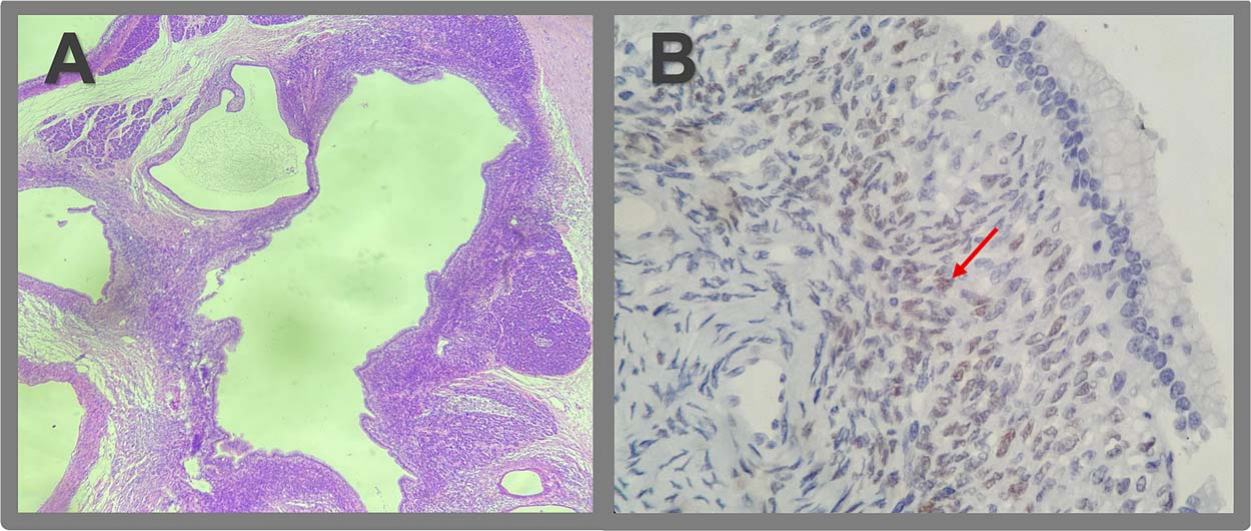
Microscopic pathologic examination. (A) Histological sections of the cystic lesion reveal a mucinous epithelial lining with regular nuclei, without atypia or architectural complexity. The capsule consists of dense connective tissue and contains areas of more cellular stroma, characterized by spindle cells without significant atypical or mitotic activity (ovarian-like stroma). A region of the capsule is replaced by granulation tissue, with evidence of prior hemorrhage and fibrosis extending to the posterior surface of the pancreas. No evidence of invasive neoplasia is observed. (B) Immunohistochemistry revealed strong positivity for estrogen receptors in the ovarian-type stromal cells, indicated by brown staining (arrow).

Gross pathology revealed a cystic lesion lined by mucinous epithelium with regular nuclei and no atypia. The capsule consisted of dense connective tissue with cellular stroma consistent with ovarian-type stroma (Figs 2 and 3). The final diagnosis was an MCN with low-grade dysplasia, measuring 3.7 cm at its largest diameter. Capsular rupture with granulation tissue and prior hemorrhage extending to the posterior pancreatic surface was documented. Resection margins were free of disease, no invasive carcinoma was found, and all lymph nodes were negative. Immunohistochemical staining demonstrated estrogen receptor positivity.

## Discussion

MCNs are rare pancreatic tumors with a clear female predominance. Their ovarian-type stroma, which expresses estrogen and progesterone receptors, supports a hormonal influence on tumor biology and explains their documented progression during pregnancy [7, 10, 11].

In a systematic review of 47 pregnant women with MCNs, 52% experienced cyst enlargement, 9% rupture, and 11% hemorrhage [6]. Although data on IVF-related cases are limited, parallels can be drawn with pregnancy. Our case aligns with the only prior report of MCN growth after gonadotropin stimulation for IVF [10], but uniquely documents cyst rupture—a potentially life-threatening complication.

The American Society for Reproductive Medicine recommends counseling women undergoing IVF about an increased risk of ovarian tumors, particularly borderline tumors [12]. A meta-analysis of 746 455 women confirmed that ovarian cancer risk persists even within the first year after IVF [12]. Extrapolating from these findings, gonadotropin-based stimulation may also accelerate MCN growth or malignant transformation.

Distal pancreatectomy remains the standard treatment for MCNs, especially those ≥4 cm, symptomatic, ruptured, or with mural nodules [3, 13]. Smaller, stable lesions without nodules may be monitored. In this case, conversion to open surgery was required due to posterior capsular rupture with associated inflammation. Histopathology confirmed low-grade dysplasia and estrogen receptor positivity, reinforcing the hormonal sensitivity of MCNs.

This case underscores the importance of considering cystic pancreatic lesions in women undergoing assisted reproduction. Hormonal stimulation may accelerate growth and increase the risk of rupture or malignant transformation.

## Conclusion

This is the first reported case of MCN rupture following gonadotropin-based ovarian stimulation for IVF. It highlights the need for awareness among reproductive specialists and surgeons regarding the potential risks of exogenous hormonal therapy in women with undiagnosed pancreatic cystic lesions. Screening and timely management of MCNs before IVF may help prevent severe complications.
